# Long-term effect of aripiprazole lauroxil on health-related quality of life in patients with schizophrenia

**DOI:** 10.1186/s12888-021-03124-2

**Published:** 2021-03-24

**Authors:** Joseph P. McEvoy, Peter J. Weiden, Paul H. Lysaker, Xiaowu Sun, Amy K. O’Sullivan

**Affiliations:** 1 Department of Psychiatry and Health Behavior, Medical College of Georgia, Augusta University, 997 St. Sebastian Way, EG-1010, Augusta, GA 30912 USA; 2 Alkermes, Inc., Waltham, MA USA; 3 Roudebush VA Medical Center, Indianapolis, IN USA; 4 Indiana University School of Medicine, Indianapolis, IN USA

**Keywords:** Aripiprazole lauroxil, Patient-reported outcomes, Health-related quality of life, Long-acting injectable antipsychotics, Schizophrenia, SF-36v2

## Abstract

**Background:**

This post hoc analysis of clinical trial data evaluated long-term, self-reported mental and physical health-related quality of life (HRQoL) scores in schizophrenia patients receiving aripiprazole lauroxil (AL), an atypical long-acting injectable (LAI) antipsychotic approved for the treatment of schizophrenia in adults.

**Methods:**

The study population included 291 stable schizophrenia outpatients enrolled in 2 consecutive long-term safety studies of AL given every 4 weeks for up to 124 weeks. HRQoL was measured using the SF-36v2® Health Survey (SF-36v2) over the course of the follow-up. The primary outcome was change in SF-36v2 mental component summary (MCS) and physical component summary (PCS) scores from baseline to 124 weeks. To contextualize these scores, descriptive analyses were conducted to compare the scores with available scores for the general population as well as for other populations with chronic medical (ie, hypertension and type 2 diabetes) or psychiatric (ie, depression) conditions.

**Results:**

Results from this post hoc analysis indicated that the mean MCS score for patients continuing AL improved significantly from baseline over 124 weeks (*P* < .05, all timepoints), while mean PCS score showed little change over 124 weeks. At baseline, patients had lower (worse) MCS scores than the normed general population, but by week 124, patients had MCS scores comparable to those in the general population. This pattern of change was not observed with PCS scores. Comparison of study MCS scores with those associated with other diseases showed that this schizophrenia cohort had lower scores than those with chronic medical conditions but higher scores than those with depression. PCS scores were higher in the study population than published scores for all reference populations at baseline and week 124.

**Conclusions:**

In this post hoc analysis, outpatients with schizophrenia who continued the LAI antipsychotic AL showed gradual and sustained improvement in self-reported mental HRQoL over several years of follow-up, whereas self-reported physical HRQoL did not change. By the end of follow-up, mental health scores of study patients with schizophrenia were comparable to those of the general population and better than those of patients with depression.

**Trial registration:**

ClinicalTrials.gov (NCT01626456 [trial registration date: June 15, 2012] and NCT01895452 [trial registration date: July 5, 2013]).

## Background

The inclusion of the patient voice in clinical research is of growing interest in the field of psychiatry as well as to regulatory agencies such as the US Food and Drug Administration [1, 2]. Attention to the patient perspective has led to research examining the effect of antipsychotic treatment on patients’ assessments of their functioning and well-being, including health-related quality of life (HRQoL) [2–5].

Although some studies have evaluated the effect of antipsychotic medication on patient-rated HRQoL, most followed patients for 1 year or less [3, 6–10]. For a chronic condition like schizophrenia, changes in HRQoL may evolve over several years. Additionally, some studies examining the relationship between antipsychotic therapy and HRQoL were conducted in patients receiving oral antipsychotics [7, 11, 12], which can be associated with poor adherence. Long-acting injectable (LAI) antipsychotics were developed to provide an alternative to oral antipsychotics for long-term treatment of schizophrenia [13]. The LAI aripiprazole lauroxil (AL), a prodrug of aripiprazole, was developed as a long-acting intramuscular formulation for the treatment of schizophrenia [14]. Efficacy and safety of AL for the treatment of schizophrenia were demonstrated in a 12-week pivotal trial [15]. Short-term AL treatment was associated with significant improvement from baseline in Positive and Negative Syndrome Scale (PANSS) total score, and continued therapeutic efficacy was observed in a 52-week extension study [15, 16].

Long-term safety studies were conducted in a cohort of outpatients with schizophrenia who were followed for up to 3.5 years while receiving monthly AL treatment [17, 18]. As part of these studies, we collected HRQoL via the SF-36v2® Health Survey (SF-36v2), which is a measure of functional health and well-being [19]. The SF-36v2 has 2 subscales, one for quality of life pertaining to mental health and the other pertaining to physical health. The current post hoc analysis examined the changes in self-reported mental and physical HRQoL in schizophrenia outpatients followed for more than 2 years while receiving AL in these studies. We then used the long-term data for 2 descriptive comparisons. First, we compared the trajectory of HRQoL scores over time with those of the normative general population. Second, we compared the cross-sectional scores at baseline and end of study for this schizophrenia sample with other cross-sectional SF-36v2 scores obtained for 2 chronic medical conditions (hypertension and type 2 diabetes) and another mental health condition (depression).

## Methods

### Study design and outcome measures

This post hoc analysis included data from patients participating in 2 consecutive phase 3, multicenter, open-label safety studies of AL for a total duration of follow-up of 124 weeks (ClinicalTrials.gov identifiers: NCT01626456 and NCT01895452) [17]. Study duration for individual patients varied by enrollment date. Baseline was defined as entry date into the first of the 2 studies.

Patient HRQoL was measured using the SF-36v2, a well-studied, widely used general instrument for collecting information from patients on their mental and physical HRQoL [19]. This instrument has been used to measure HRQoL in many clinical conditions and is normed to the US general population [20, 21]. The SF-36v2 has demonstrated validity and reliability in patients with schizophrenia [19].

The items of the SF-36v2 assess 8 domains of functional health and well-being: physical functioning, role limitations due to physical health, bodily pain, general health, vitality, social functioning, role limitations due to emotional health, and mental health. Weighted aggregates of these 8 domain scores are used to calculate 2 summary scores: the mental component summary (MCS) score and the physical component summary (PCS) score [22]. The SF-36v2 domains and summary scores are all converted to a T-score (mean: 50; SD: 10) that was standardized using a US general population normative sample derived from responses to a 2009 online normative survey conducted by QualityMetric [19]. Higher scores indicate better health outcomes for all domains and summary measures.

Patients in the AL trials completed the SF-36v2 at baseline, 6 times during the first safety study (weeks 4, 12, 20, 32, 48, and 52 or end of treatment) and 6 times during the second safety study (every 12 weeks and at the end of the study). For the post hoc primary study outcome, we evaluated the change in mental and physical HRQoL as measured by the SF-36v2 MCS and PCS scores from baseline to week 124 using least squares estimates. To contextualize the baseline and endpoint scores for our outpatient schizophrenia cohort, we descriptively compared baseline and week 124 SF-36v2 MCS and PCS scores for patients in the AL trials with those published for the general population and for 3 chronic conditions: the medical conditions of hypertension and type 2 diabetes and the mental health condition of depression (defined as major depression and/or dysthymia) [19]. These conditions were chosen for comparison because they are common in the general population.

### Patients

Eligible patients were aged 18 to 70 years at study entry and were clinically stable, with a primary diagnosis of schizophrenia (*Diagnostic and Statistical Manual of Mental Disorders*, Fourth Edition, Text Revision) [15]. Further details of the design, inclusion and exclusion criteria, and primary safety results for the first of the 2 studies in our analysis are reported elsewhere [17].

### Treatments

Patients received 1 of 2 fixed-dose regimens of AL: 441 mg or 882 mg every 4 weeks. AL dose assignment has been described previously [17]. No dose adjustments of AL were permitted during the follow-up period.

### Statistical analysis

The primary outcome of this post hoc analysis was the change in patients’ self-reported HRQoL (as measured by SF-36v2 MCS and PCS scores) from baseline to 124 weeks (least squares estimates). The post hoc analysis population included all patients in the first safety study full analysis set who completed the study and then entered and completed at least 1 HRQoL assessment in the second study.

Descriptive statistics are reported, including frequencies and percentages for categorical variables and means (SDs) for continuous variables. For the SF-36v2 measures, mixed models for the change from baseline for MCS and PCS were fit with observed data after controlling for baseline score and geographic region. The least squares estimates for change score from baseline were obtained for mixed models. All analyses were performed using SAS 9.4 (SAS Institute, Cary, NC, USA).

Minimal important differences (MIDs) can be useful in interpreting SF-36v2 summary scores, as they provide a context for understanding score differences that extend beyond the evaluation of statistical significance. An MID is the smallest score difference between 2 independent groups that is perceived as clinically important or meaningful [23]. Threshold values representing MIDs have been developed for the SF-36v2 domain and summary component scores based on data collected during the 2009 norming study [19]. The MID for the PCS is 2 points, and the MID for the MCS is 3 points [19]. When mean score differences between 2 groups exceed these thresholds, the score difference can be interpreted as clinically meaningful; score differences that do not exceed these thresholds are likely not meaningful.

As a secondary outcome in this post hoc analysis, baseline and week 124 SF-36v2 MCS and PCS scores for the AL population were descriptively compared with those for the general population and individuals with hypertension, type 2 diabetes, and clinical depression obtained from Maruish et al. [19].

## Results

### Patients

**Fig. 1:**
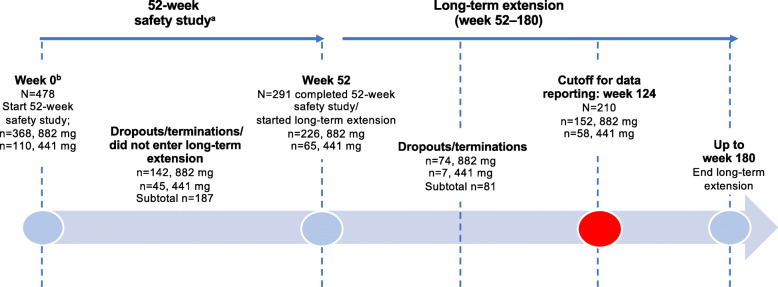
Patient timeline and disposition. ^a^Nasrallah HA, et al. [17]. ^b^The week 0 timepoint corresponds with baseline for this analysis

Figure 1 shows patient disposition, and Table 1 lists the demographic and baseline characteristics of the 291 patients with schizophrenia who composed the study population. The patient population was 53.6% male and 69.8% white; 65.3% of patients were from Europe, 22.3% were from Asia, and 12.4% were from North America. Mean (SD) age at baseline was 38.6 (11.5) years; 88.7% of patients were under 55 years of age. Mean PANSS scores at baseline (mean total score: 58.9 [SD: 13.4]) were consistent with mild illness [24].

**Table 1:** Baseline Patient Characteristics

Characteristic	***N*** = 291
**Age, mean (SD), y**	38.6 (11.5)
**Sex, n (%), male**	156 (53.6)
**Race, n (%)**	
White	203 (69.8)
Asian	65 (22.3)
Black or African American	22 (7.6)
Native Hawaiian or Pacific Islander	1 (0.3)
**Ethnicity, n (%)**	
Hispanic or Latino	11 (3.8)
Not Hispanic or Latino	280 (96.2)
**Region, n (%)**	
Asia	65 (22.3)
Europe	190 (65.3)
North America	36 (12.4)
**PANSS total score,**^**a,b**^ **mean (SD)**	58.9 (13.4)

*PANSS* Positive and Negative Syndrome Scale

^a^The PANSS is a 30-item scale with item scores ranging from 1 (not present) to 7 (extreme). The score range is 30–210, with higher scores indicating more severe symptoms [24]

^b^Baseline PANSS total score was based on 290 patients who had evaluable data

### Outcomes

#### Primary outcome: trajectory of mental and physical HRQoL

**Fig. 2:**
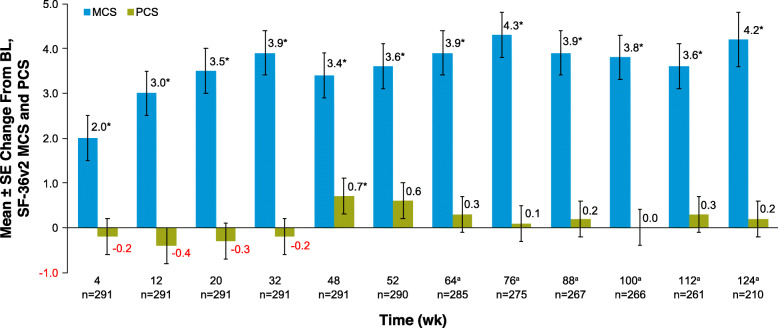
Longitudinal mental and physical HRQoL. Data are least squares mean estimates of change from baseline (ie, week 0); timepoints are not shown to scale. ^a^After 52 weeks, patients switched to the long-term extension study and completed the SF-36v2 every 12 weeks. *P < .05 vs week 0 baseline. BL, baseline; HRQoL, health-related quality of life; MCS, mental component summary score; PCS, physical component summary score; SF-36v2, SF-36v2 Health Survey

Results from least squares estimates from mixed models in this post hoc analysis indicated that mental HRQoL as measured by the mean MCS score improved significantly over 124 weeks compared with baseline in those continuing treatment (change was significantly higher at *P* < .05 at all timepoints vs baseline; Fig. 2). Physical HRQoL showed little change over the follow-up period, with the exception of week 112 when the mean PCS score in the study population was significantly higher compared with baseline (*P* < .05) (Fig. 2).

#### Comparison of study population and general population

**Fig. 3:**
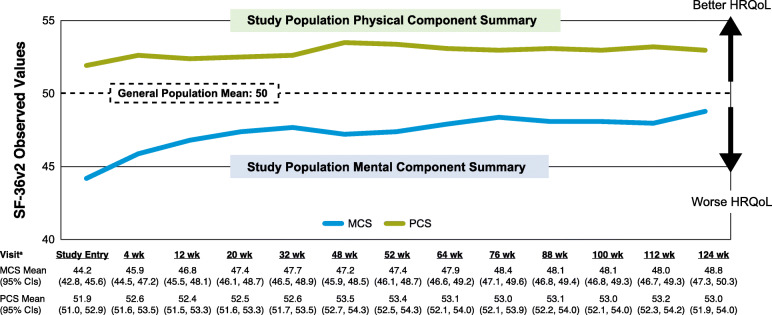
Trajectory of self-reported HRQoL during continuation treatment with aripiprazole lauroxil. ^a^After 52 weeks, patients completed the SF-36v2 every 3 months, where 1 month = 28 days − 7/+ 14 days. HRQoL, health-related quality of life; MCS, mental component summary score; PCS, physical component summary score; SF-36v2, SF-36v2 Health Survey

Figure 3 displays the mean MCS and PCS scores from baseline through week 124 in relation to the general population mean. At baseline, the study population’s mean MCS score was lower than the general population mean score (mean: 44.2 [SD: 10.4] vs 50); this difference clearly exceeded the 3-point MID; however, beginning at week 20, mean MCS scores in the study population were not substantially different (ie, were not greater than the MID) from those in the general population. At baseline, PCS scores in the study population were not substantially different from those in the general population (mean: 51.9 [SD: 7.2] vs 50); however, beginning at week 4, PCS scores in the study population were higher than those in the general population (ie, differences in mean PCS scores between the study population and general population norm exceeded the 2-point MID).

#### Comparison of study population and patients with other conditions

**Fig. 4:**
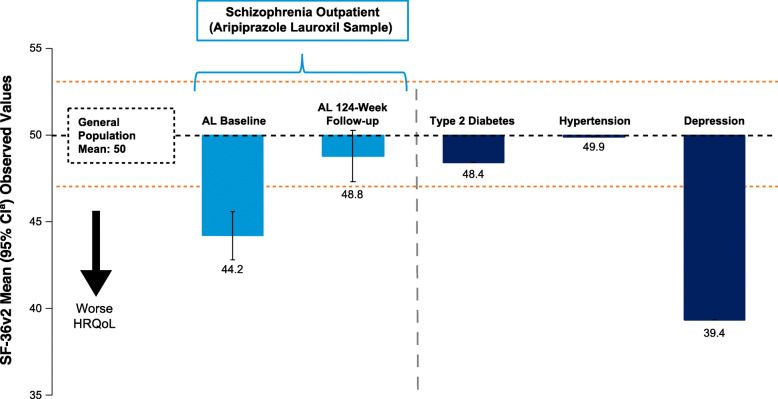
Baseline and follow-up schizophrenia mental health HRQoL (mean MCS scores) compared with other chronic conditions. ^a^95% CIs are presented for AL means only; 95% CIs were not available for the other populations. Orange dotted lines indicate minimal important differences relative to the general population mean for MCS scores. AL, aripiprazole lauroxil; HRQoL, health-related quality of life; MCS, mental component summary score; SF-36v2, SF-36v2 Health Survey. Data for type 2 diabetes, hypertension, and depression are from Maruish et al. [19]

Figure 4 shows comparisons of the mean MCS scores for the study population at baseline and 124 weeks with published means for patients with other common medical conditions.

At baseline, the mean MCS score for the study population (44.20) was lower than the scores for patients with hypertension (49.93) or type 2 diabetes (48.42); differences between these MCS scores for the study population and the 2 conditions exceeded the 3-point MID threshold (Fig. 4). At week 124, the mean MCS score for the study population was not meaningfully different relative to the mean scores for the hypertension and type 2 diabetes populations (based on the MID for MCS).

At baseline, the study population’s mean MCS score (44.20) was higher than that of the patients with clinical depression (39.37) and exceeded the 3-point MID, indicating better mental HRQoL in the study population compared with the reference clinical depression population (Fig. 4). At week 124, the mean MCS score for the study population remained higher than that of the clinical depression population.

At baseline and 124 weeks, the mean PCS score for the study population was higher than the scores for patients with the 3 aforementioned medical and psychiatric conditions, indicating better physical HRQoL for the study population both at baseline and 124 weeks (data not shown).

## Discussion

This post hoc study examined the trajectory of change in self-reported HRQoL in stable patients with schizophrenia maintained on therapy with AL over 124 weeks measured by the SF-36v2, a commonly used instrument for measuring function and well-being. In addition, we descriptively compared clinical trial results with publicly available normed values from the general population as well as patients with 3 chronic conditions (hypertension, type 2 diabetes, and depression). To provide further context to the interpretation of differences between the schizophrenia clinical trial population and the external populations, we used threshold values representing MIDs.

Our main finding is that mental HRQoL improved and was maintained over the course of 2 years compared with baseline scores in this population of patients with schizophrenia. This change does not seem to be an artifact of acute symptoms at baseline, given that the mean baseline PANSS score indicated that overall severity of illness was mild. Based on the MID for MCS, the baseline mean MCS score in the study population was lower than that in the general population, but by the end of the follow-up period over 2 years later, scores from the patients with schizophrenia did not differ from the normative general population scores. Some variability between post-baseline timepoints was noted in mean MCS and PCS scores; however, the magnitude of that variation was less than the MID of 3 points for the MCS and 2 points for the PCS and, therefore, is likely not clinically meaningful. In contrast, baseline PCS scores in the study population already resembled those in the general population at baseline and did not substantially change over time. Further, at week 124, mental health scores (MCS scores) in the study population were comparable to those among patients with hypertension or type 2 diabetes and were well above those among patients with depression (based on the MID). This finding is in line with findings from studies showing that major depressive disorder, as well as symptoms of depression in patients with schizophrenia, are associated with poor quality of life and that patients with depression have significantly poorer quality of life than either healthy populations or patients with other chronic diseases including hypertension [4, 25–27].

Our results suggest that continuing to treat stable patients with schizophrenia with the LAI antipsychotic AL is associated with improvements in self-reported HRQoL. Previously published studies of data from our cohort demonstrated the durability of remission with AL therapy [16] as well as the drug’s long-term safety and tolerability [17]. The tolerability of AL may contribute to patients rating their physical HRQoL (mean PCS score) similarly to that of the general population mean. The clinical observation that patients with schizophrenia underreport physical symptoms [28, 29] also may contribute to this finding.

Our study findings for SF-36 MCS scores for a schizophrenia cohort are generally consistent with those of other studies. A recent analysis of data from an international observational study aimed to provide a better understanding of the use of SF-36 and a second HRQoL scale, the EuroQol-5 Dimension questionnaire, in patients with schizophrenia by analyzing the correlation between these general scales and disease-specific instruments and disease characteristics, as well as the correlations among the instruments themselves [30]. Sociodemographic and clinical correlates were analyzed, along with their consistency with condition-specific scale scores (PANSS and Schizophrenia Quality of Life Scale [SQLS]). Among 1379 patients with schizophrenia, the mean PANSS score was 77.98 and the mean MCS score was 40.68. Among the correlates of MCS were PANSS positive and negative dimension ratings (where higher PANSS scores were associated with lower MCS scores) and PANSS depression ratings (associated with lower MCS). The SF-36 MCS was highly correlated with the SQLS, showing internal consistency with symptom-specific HRQoL.

Several studies in the literature have examined the effects of new treatments on HRQoL in schizophrenia but over considerably shorter durations than in the current study. Our study has several strengths relative to the existing literature, the latter of which predominantly report HRQoL results associated with oral or LAI antipsychotics for up to 1 year [3, 8, 31, 32]. We report an extended follow-up period (> 2 years) for patients with schizophrenia, measured HRQoL with a validated patient self-reported instrument, and included patients on an LAI agent, which serves to reduce adherence as a confounder.

The current analysis has several limitations. The inclusion of a subgroup of patients who successfully completed the 12-week pivotal efficacy and safety study may limit the generalizability of the results to a broader population of patients with schizophrenia, as it applies to a more stable cohort of patients. The attrition of patients who had poor tolerability or lack of clinical efficacy during the 124 weeks, as summarized in a safety analysis that included this patient population [18], may also bias these long-term outcomes, as patients who continued were likely to be those who had a positive treatment response without major tolerability issues. Results from this study may not generalize to patient populations excluded from study enrollment, such as those with treatment-resistant schizophrenia or those experiencing their first episode of schizophrenia who are not clinically stable.

The study may also have been limited by the use of a generic (ie, non–disease-specific) measure rather than one that specifically addresses quality-of-life issues facing persons with schizophrenia. The SF-36v2, unlike tools designed for populations with serious mental illness, will not detect certain issues, such as stigma, affecting quality of life for these patients. These disadvantages are offset by the notable advantages shown here; specifically, the SF-36v2 is normed to the general population, and SF-36v2 HRQoL data are available for patients with other diseases, a feature that allows the comparisons presented in this paper. Because the SF-36v2 is a self-reported measure, its validity in patients with an illness like schizophrenia, which can affect insight, may be questioned. However, at least 2 previous studies have demonstrated that self-reported HRQoL in patients with schizophrenia is consistent over time and correlates well with clinician ratings for patients with schizophrenia who are not severely ill [5, 12].

Additional limitations of the current analysis include the open-label and post hoc design, as well as the absence of a placebo control group. Comparing SF-36v2 scores from our study patients with normative data from other populations provides points of reference for a qualitative picture of HRQoL with AL; however, without a placebo control, possible effects on HRQoL of factors related to study procedures other than AL treatment cannot be discounted. Further, normative data were based on US population data; therefore, variability may exist between characteristics in the general population and those in the study population, as the latter was internationally based. Another limitation in the descriptive cross-disease comparisons is related to generalizability of data from a cohort from a single clinical trial to the broader disease population of schizophrenia (the same can be said for the data used for the other chronic conditions as well). Therefore, these data should not be used as a definitive comparison. To our knowledge, this is the first study of the SF-36v2 scale in a long-term clinical trial in which HRQoL of patients with schizophrenia was compared with that of patients with other diseases, and the purpose of these comparisons was to offer readers more qualitative perspectives.

## Conclusions

Our major finding was that ongoing treatment with the atypical LAI antipsychotic AL was associated with significant improvements in self-reported mental health quality of life that were sustained over a follow-up period lasting over 2 years. The pattern of MCS score improvement occurred in large part within the first 4 weeks of treatment and was maintained during the observation period. By week 20, mean patient scores were similar to normative scores of the general population. Physical HRQoL was reported to be within the range of the general population norms and was sustained without much change — either improving or worsening — throughout the same follow-up period. In sum, results of this study indicate that the LAI antipsychotic AL improves (and maintains) mental HRQoL in stable patients with schizophrenia.

In the first study of its kind, we compared SF-36v2 MCS and PCS scores in this population of patients with schizophrenia to those in patients with 2 medical conditions (hypertension and diabetes) and another mental health condition (depression). The mental health scores of the schizophrenia population started lower (worse) than the scores of the population with other medical conditions but better than the scores of the population with depression. These comparisons help to anchor the baseline data to other conditions, something that has not frequently been done in quality-of-life literature in schizophrenia cohorts.

## Data Availability

The data collected in this study are proprietary to Alkermes, Inc. Alkermes, Inc., is committed to public sharing of data in accordance with applicable regulations and laws, and requests can be submitted to the corresponding author.

## Abbreviations

AL: Aripiprazole lauroxil
HRQoL: Health-related quality of life
LAI: Long-acting injectable
MCS: Mental component summary
MID: Minimal important difference
PANSS: Positive and Negative Syndrome Scale
PCS: Physical component summary
SF-36v2: SF-36v2 Health Survey
